# First referral hospitals in low-resource settings: a narrative review of expectations for clinical service provision

**DOI:** 10.1093/heapol/czaf021

**Published:** 2025-03-31

**Authors:** Tamara Mulenga Willows, Rosanna Mazhar, Suraj Bhattarai, Chit-Su Tinn, Nadine Misago, Jean Jacque Roger Ikuzwe, Mike English

**Affiliations:** Health Systems Collaborative, NDM Centre for Global Health Research, Nuffield Department of Medicine, University of Oxford, Peter Medawar Building, South Parks Rd, Oxford OX1 3SY, United Kingdom; Wolfson Institute of Population Health Charterhouse Buildings, Queen Mary University of London, London EC1M 7BA, United Kingdom; Health Systems Collaborative, NDM Centre for Global Health Research, Nuffield Department of Medicine, University of Oxford, Peter Medawar Building, South Parks Rd, Oxford OX1 3SY, United Kingdom; Global Health Research & Medical Interventions for Development (GLOHMED), Kathmandu, Nepal; London School of Hygiene & Tropical Medicine, London WC1E 7HT, United Kingdom; Centre for Tropical Medicine and Global Health, Nuffield Department of Medicine, South Parks Rd, University of Oxford, Oxford OX1 3SY, United Kingdom; Interdisciplinary Research Group in Public Health, Doctoral School, University of Burundi, Bujumbura, Burundi; Independent Researcher, Kigali, Rwanda; Health Systems Collaborative, NDM Centre for Global Health Research, Nuffield Department of Medicine, University of Oxford, Peter Medawar Building, South Parks Rd, Oxford OX1 3SY, United Kingdom; Health Services Unit, KEMRI-Wellcome Trust Research Programme, Nairobi, Kenya

**Keywords:** first-level hospitals, referral hospitals, first-referral hospitals, district hospitals, hospital health systems

## Abstract

First referral hospitals (FRHs) have an important role to play in helping many countries achieve ‘Health for All’. However, their specific role and the clinical services they are expected to provide to achieve this are evolving. To explore this issue further, we undertook a narrative review to examine the clinical service expectations of FRHs outlined in academic and policy literature, which identified a total of 404 FRH service expectations. At a global level, some categories of services provide extensive specific service recommendations, likely resulting from historical priorities and the influence of vertical programming and professional interests. However, in several important areas we identified few or no recommendations. At the level of individual country case studies undertaken through this review, FRH clinical service recommendations within available policy documents vary considerably. Our findings suggest a disconnect between the ambition for FRH and the difficult, context-specific decision-making needed at the national level on the role of FRHs as a service delivery platform within integrated health systems helping countries achieve universal health coverage.

Key messages

- Since the Declaration of Alma Ata, formal normative guidance about the expected role or services that should be provided by platforms created to support primary care at the first referral hospital (FRH) level has been limited.
- Expectations or suggestions for clinical services that could be provided at the FRH have however been published in many service or discipline-specific reports, without consideration for how they might collectively and cumulatively impact what is expected of an imagined FRH.
- In this review, we attempt to collate the range of expectations for service delivery at FRH in the modern era and provide an opportunity for global and national policymakers to reflect on the role of FRHs in relation to wider health systems, with an aim to promote discussion on the role FRH might play as a clinical service platform in different contexts to support primary care and universal health coverage.

## Introduction

Since the Declaration of Alma Ata, first referral hospitals (FRHs) have fallen off the global agenda and there has been a dearth of guidance outlining what role they should play today in helping countries achieve ‘Health for All’. The Alma Ata Declaration of 1978 promoted primary health care (PHC) as the key to attaining ‘Health for All’ and recognized key roles of FRHs (first-level hospitals) in achieving these goals (WHO 1978). In 1985, a WHO committee agreed that hospitals should be fully involved in both the planning and delivery of primary healthcare at the district level. The interdependence between hospitals and communities was emphasized, and FRHs were tasked with providing a ‘fully comprehensive range of promotive, preventive, curative, and rehabilitative health activities’ to reach the communities they serve (WHO 1987).

In 1992, the World Health Organization (WHO) published guidance on district hospitals (the contemporary term for FRH) that defined their requisite characteristics and formalized the expansion of their role beyond curative care to one that also supports the delivery of health services at the community level (WHO 1992). Shortly after, in 1996, WHO’s regional office for the Pacific published a guideline that further outlined both the planning and design requirements for district hospitals in that region, stating that district hospitals should be able to serve 85%–95% of a district’s health needs (WHO 1996). In this report, we use the term FRH as a more general term rather than district hospital (for a full discussion see reference) (Mazhar et al. 2024). The 1992 WHO report provided some guidance on FRH’s expected clinical service and clinical support departments (Table 1), while the 1996 report refrained from providing a prescriptive list of expected clinical services, anticipating that this would reflect countries’ varying epidemiological profiles, population sizes, and geographic and climatic conditions (WHO 1992, 1996).

**Table 1.** WHO 1992 Technical Report Series recommendations for service departments for the district hospital and classification of DCP3 essential interventions for first-level hospitals

WHO		DCP3
Department	Service	Maternal and newborn health Surgery Child health Reproductive health HIV Cancer Tuberculosis Adult febrile illness Cardiovascular disease Musculoskeletal Congenital disorders Injury Rehabilitation Palliative care Pathology
Clinical		
Medicine Surgery Paediatrics Obstetrics/Gynaecology Dentistry Orthopaedic surgery Otorhinolaryngology Neurology Psychiatry	Essential Essential Essential Essential Essential Essential Essential Essential Essential	
Clinical support		
Anaesthesia Radiology Clinical Laboratory Pathology Rehabilitation	Essential Essential Essential Optional Optional	

The most current specific guidance on expected clinical services for FRH is published in the 2015 Disease Control Priorities third edition (DCP3), which defines essential health service packages for PHC and FRH based on their cost-effectiveness as part of universal health coverage (UHC) (Watkins et al. 2017). This analysis recommends 58 minimum services or interventions that should be provided by FRH (Table 1), which DCP3 defined as a ‘facility with the capacity to perform surgery and provide inpatient care, outpatient specialist care and routine pathology services that cannot be feasibly delivered at lower levels’ (Watkins et al. 2017). However, this guidance was not intended as a comprehensive description of service expectations for FRH as integrated health delivery platforms in low-resource settings.

More recently, in 2020, the WHO launched the UHC Compendium database which is a steadily expanding list of 460 interventions linked to 3602 specific actions as of 1 August 2023 (WHO 2023a). These interventions and actions are linked to 29 health programme areas that vary from the disease-specific (e.g. HIV) to much wider service areas (e.g. maternal and newborn health) (WHO). This project is ongoing and is designed ‘to assist countries in making progress towards Universal Health Coverage (UHC)’ and ‘provides a strategic way to organize and present information and creates a framework to think about health services and health interventions’. The aim of this approach is to present health services and interventions across ‘the full spectrum of promotive, preventive, diagnostic, resuscitative, curative, rehabilitative, and palliative services, as well as a full complement of intersectoral interventions’. It also aims to ‘provide rapid one-stop access to supporting evidence, associated human and material resource inputs’. While it does not currently disaggregate services or interventions by suggested service delivery level, this is planned in the future (WHO). The long-term aim of the UHC Compendium is to support countries developing UHC packages and link selected interventions to service delivery platforms through context-derived prioritization as part of an overall strategy for Integrated Primary Health Care Systems that include and envisage broad roles for hospitals in support of primary care (World Health Organization and the United Nations Children’s Fund (UNICEF) 2020).

Our interest, and the subject of this report, is to explore existing expectations for services or interventions that experts suggest FRH might deliver as identified in contemporary literature. This includes a growing set of subject-specific recommendations from authoritative sources such as discipline-specific expert groups. Our aim is to highlight the scope of clinical service expectations of FRHs as articulated in such sources, based on a ‘sum of the parts’ approach, and explore what these tell us about current expectations for FRH as a service delivery platform in support of PHC. We do this by reviewing and synthesizing published recommendations from global policy and academic literature. We complement this by documenting and then contrasting this summative approach to FRH service expectations to the descriptions of FRHs in selected low- and middle-income country case studies as examples of different contexts. In this paper, we use the term low-resource countries to refer to a broad range of countries that have varying economies, including high-income countries, but with public health systems that are unable to meet the needs of populations due to resource constraints within their health systems (Van Zyl et al. 2021). In demonstrating the extent and increasing range of expectations, we hope to re-instigate reflection on the role and development of FRHs in low-resource settings as part of integrated health systems.

## Materials and methods

Our review focused on the clinical service expectations for FRH in the public sector, in low-resource settings, and in contemporary literature. Given the broad nature of this question, we undertook a narrative review. Our information sources include academic literature, policy literature, and seven country case studies examining national FRH-related policies.

### Academic literature search strategy

We divided the academic literature portion of our search strategy into three parts with a focus on literature targeting public health systems with low availability of resources availability. Each stage of our structured process is outlined in Supplementary Appendix 1 along with explicit inclusion and exclusion criteria for articles. An information specialist searched the following databases on 3 August 2022 with no limits for language or publication dates: Ovid Embase; Ovid MEDLINE; Ovid Global Health; Ovid PsycINFO; Ovid AMED; EBSCOhost CINAHL; the Cochrane Database of Systematic Reviews; and the Cochrane Central Register of Controlled Trials (specific search terms included in Supplementary Appendix 1). For the sake of completeness, we supplemented this structured search strategy with (i) select articles held by the senior reviewer in a pre-existing repository on the subject and (ii) purposively screening the *Lancet* Commission and the *Lancet* Series papers.

All identified articles were title and summary screened using Rayyan software for the structured academic review, and directly from the *Lancet* Commission and Series websites. We divided articles selected for full-text review between two reviewers to determine suitability for inclusion. These were cross-checked and underwent a second round of exclusions under the guidance of a senior reviewer (M.E.) to derive a final list of included articles.

### Policy literature search strategy

We relied on WHO and United Nations Population Fund (UNFPA) websites’ health topic lists for our policy literature review (UNFPA 2023, WHO 2023b). For each health topic, one reviewer scanned through the indexed list of publications to identify guidelines/normative documents, excluding publications >30 years old on the assumption that these would be outdated and likely replaced by more recent documents. For each document identified, we searched for any use of the terms ‘hospital’ and ‘facility’ and read contextually to identify any specific recommendations regarding clinical service expectations at the FRH level. We purposively included selected documents that did not come up in our search, but we now contain recommendations targeting FRH, including the pocketbook of hospital care for children and the integrated management of adolescent and adult illness district clinician manual. For these two documents, we inferred expected FRH services from topic-specific headings and subheadings and case management guidelines. We excluded content, such as from clinical algorithms, which simply stated ‘refer to hospital’ but that provided no service-specific guidance for the FRH itself. As documents were duplicated across multiple WHO health topics, we did not document the number of documents screened for this part of our review.

We additionally searched global medical specialty professional organizations’ websites for potentially relevant publications, which we reviewed using the same approach taken for WHO and UNFPA publications, to identify any relevant professional organizations’ recommendations.

### Analysis framework

To enable unified data extraction, analysis, and presentation of the review findings, we developed service categories through an iterative process informed by categorizations observed in national health service packages that are largely consistent with, but fewer than, the health programme areas used in the UHC Compendium. Our resulting ‘review categories’ are not necessarily synonymous with FRH clinical departments. These formed the basis of our framework for analysis (Table 2).

**Table 2.** Review categories

Review categories	Notes
Emergency, trauma, and acute care	All health services related to the management of health emergencies and accidents that may or may not result in physical trauma.
Advanced medical care	All health services related to the care of advanced medical and surgical conditions. This includes intensive care units, high-dependency units, critical care units, and other advanced care departments.
Ear, nose, and throat (ENT)	All ENT-related services (inpatient and outpatient).
Eye health	All eye care-related services (inpatient and outpatient).
Oral health	All oral hygiene/dental-related services.
Surgery & anaesthesia	All health services related to any surgery, including anaesthesia.
Female reproductive health	All health services (inpatient and outpatient) related to female reproductive health outside maternal care, including family planning, gynaecological services, cervical cancer and screening.
Maternal health	All health services (inpatient and outpatient) related to pregnancy, delivery, and post-natal care.
Newborn health	All health services (inpatient and outpatient) related to the care of newborns.
Child health	All health services (inpatient and outpatient) where recommendations are specific to the needs of children and can include newborns if not specific to that group.
Adolescent health	All health services (inpatient and outpatient) where recommendations are specific to the needs of adolescents.
Elderly care	All health services where recommendations are specific to the needs of the elderly.
Mental health	All health services (inpatient and outpatient) related to the management of mental health conditions.
Non-communicable diseases (NCDs) and cancer	All health services (inpatient and outpatient) related to the management of all NCDs and cancer, irrespective of severity (excludes mental health).
Communicable diseases	All health services (inpatient and outpatient) related to the management of all communicable diseases, irrespective of severity, except for TB/HIV.
Tuberculosis (TB)/human immuno-deficiency virus (HIV)	All TB- and HIV-related services (inpatient and outpatient).
Nutrition and malnutrition	All health services related to supporting optimum nutrition in children and adults and the management of malnutrition.
Gender-based violence	All services related to meeting the health needs of survivors of gender-based violence.
Laboratories	All laboratory-based health services that assist in the diagnosis and monitoring of health conditions.
Blood bank services	All services related to the supply of blood and blood products.
Pharmacy	All pharmacy-related services.
Radiology/imaging/electrophysiology	All imaging-related health services that assist in the diagnosis and monitoring of health conditions.
Palliative care	All health services related to care of people with terminal conditions and end-of-life care.
Rehabilitation	All health services related to the rehabilitative recovery from medical or surgical conditions. This includes physiotherapy and occupational therapy.

### Literature review data extraction and analysis

All included academic articles and policy documents were split between two reviewers to extract data on FRH-specific clinical service recommendations using an Excel-based tool, assigned to the above-described review categories. Once extracted, all data were cross-checked by the other reviewer to ensure a standardized approach and to review the validity of inclusion, with any disagreements resolved through discussion. Upon completing the review, data from all literature sources were merged for narrative synthesis and descriptive analysis (Azen and Walker 2021). We aimed to avoid duplication of recommendations that featured in more than one document source and that might be associated with more than one service category. For example, we aggregated laboratory diagnostic recommendations that might be made in disease-specific documents into the ‘laboratories’ category. However, when a conceptually similar recommendation appeared as an important element of more than one service category, it was retained in both categories. For example, the treatment for severe sepsis is included in maternal, neonatal, child, and adult service categories. While our focus was on identifying specific service recommendations, during data analysis we identified certain broader statements which we termed ‘organizational arrangements’, these are summarized in narrative statements linked to service categories.

### Country case studies

To supplement global-level findings and explore how FRHs seem to be defined at a country level, we studied FRH policies in selected countries with lower-resourced public health systems. Given language limitations within our team, and our reliance on in-country professionals to access relevant government documents, countries were selected purposively. While attempts were made to represent different world regions, the selected countries (Rwanda, Sri Lanka, Nepal, Burundi, South Africa, Myanmar, and Vietnam) were not intended to be representative of all low-resource settings and are not presented as such. For each country, we undertook a health system literature review, determined the place or nature of FRHs within the health system, and extracted information on expected FRH service categories from national service packages, using an MS Word template (Supplementary Material 1). Where possible, researchers in the countries we explored provided additional information and/or translation for documents not available in English. We transferred relevant information into an Excel sheet for comparative analysis, making use of the review categories in Table 2 for ease of analysis and presentation. To further explore variability in specifications, we examined the maternal care service category in more detail as this was clearly indicated as a function of FRHs in every case study country.

## Results

### Search results

We screened 2703 academic papers, and of these, 126 were full-text reviewed. Among these, just 16 (0.6%) included recommendations for FRH clinical services, most of which came from the *Lancet* Series and Commissions as shown in Table 3 (see Supplementary Material 2 for the list of included academic papers). We reviewed 1096 policy documents and included 66 (6%) in our review, among which 94% were from the WHO (see Supplementary Material 3 for the list of included policy documents). We reviewed 48 professional organization websites and their publications but did not find any publications with relevant content. We included a total of seven countries as example cases in our review.

**Table 3.** Summary of academic and policy review search results

Source	Number of titles screened	Number of full-text reviewed	Number included in review (% of full text)
Academic literature			
Structured Literature Review	680	39	1 (2.6%)
The *Lancet* Commissions	85	25	5 (20%)
The *Lancet* Series	1922	46	8 (17.4%)
Repository Literature Review	16	16	2 (12.5%)
Total academic literature	2703	126	16 (12.7%)
Policy literature			
WHO	N/A	925	61 (5.5%)
UNFPA	N/A	171	5 (2.9%)
Professional organizations	N/A	0	0 (0.0%)
Total policy literature	N/A	1096	66 (6.0%)

### Academic and policy review findings

**Figure 1.**
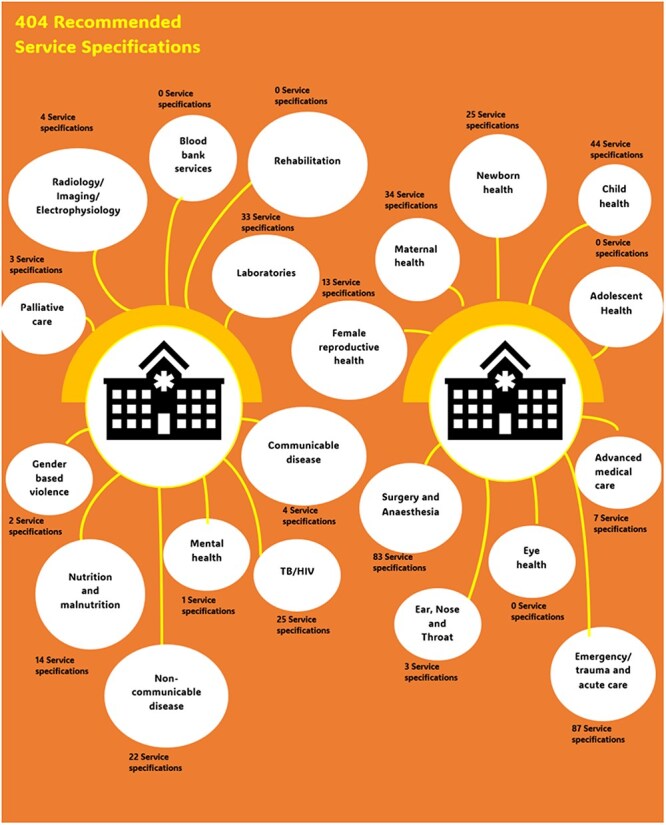
Count of FRH clinical service recommendations from academic literature and policy documents, by category.

Overall, our review of academic and policy sources revealed a total of 404 FRH clinical service recommendations for hospitals in low-resource settings, spanning 21 of our 24 review categories. The distribution of these by review category is outlined in Fig. 1, while the full list is provided in Supplementary Appendix 2. Interestingly, among the documents we reviewed, there was a complete absence of specific FRH service recommendations for elderly care, oral health, and pharmacy services (although there are recommendations for essential drugs). There were hardly any internal medicine recommendations and those included were in relation to communicable disease complications. Some services such as HIV-related recommendations are featured in maternal health, newborn health, child health, and communicable diseases recommendations. The seven service areas of child health, emergency and trauma care, laboratories, maternal health, newborn health, surgical care, and TB/HIV accounted for the largest Count of FRH clinical service recommendations from academic literature and policy documents, by category proportion of specific recommendations (*n* > 25 each). We only identified one recommendation targeting FRH for eye health, adolescent health, blood bank services, and rehabilitation services across our sources.

Among the service recommendation categories identified, we found limited guidance on what the minimum requirements should be within each category. We also did not encounter unified service standards, as illustrated in Supplementary Appendix 2. Instead, services are subcategorized based on medical understanding and pathophysiology.

### Country case study findings

#### Classifications

One of the most notable findings from the country case studies is the difference in classifications and degrees of refinement of recommendations for clinical services to be delivered by FRH, as shown in Supplementary Appendix 3. For example, Myanmar groups services under slightly broader categories, including essential emergency, medical, surgical, obstetric, paediatric, orthopaedic, anaesthesia, mental health, and dental health care, followed by detailed case management guidelines. Sri Lanka’s Essential Health Service Package provides more detailed/granular service expectation checklists by service delivery platform, based on a life course approach. Meanwhile, Rwanda’s guideline features details of individual interventions per service delivery platform, including information on staff requirements for these. Approaches to groupings and subdivisions varied too, for example, Sri Lanka and Nepal are the only two countries for which elderly care is a standalone heading (elderly care and geriatric health services, respectively). In South Africa, ‘services for conditions of older persons’ are listed under the broader heading ‘medicine’.

#### Scope of services

We also observed differences in the scope of FRH services. Maternal health, child health, emergency and trauma care, and surgical care services feature in all country case studies’ guidelines, and mental health, infectious diseases, and radiology, laboratory, and oral health were covered in six out of seven countries’ recommendations for care at FRH. However other service categories are less universal. Vietnam is the only country that lists oral health and eye health as distinct service classifications related to FRH, albeit with limited detail, while Sri Lanka is the only country that separates out gender-based violence and adolescent health services as distinct FRH service categories. Finally, certain countries list expected FRH services that reflect particular country contexts, such as ‘herbal medicine’ in Vietnam and ‘Ayurveda and other traditional health services’ in Nepal.

Differences in the content of services are also apparent *within* a specific heading as exemplified by each country’s specific content related to ‘maternal health’ (see details in Supplementary Appendix 4). For Vietnam and Burundi, we did not find a breakdown of expected FRH services within ‘maternal health’ in policy documents. Among the remaining five countries that did provide more granular recommendations, there were many similarities such as a focus on antenatal and delivery care. However, only two countries specifically included postnatal care recommendations (Sri Lanka and South Africa) although neither outlined detailed content therein. While all five mention management of normal labour, just four specifically list FRH services consistent with comprehensive emergency obstetric and newborn health (including provision of caesarean section). When we compared the maternal health clinical service recommendations extracted from the literature (in Supplementary Appendix 2) with those found in country case studies (Supplementary Appendix 4), it was evident that there was a greater degree of refinement in the former compared to the latter.

#### Incorporation of the scope of global recommendations at the country level

We observed that Sri Lanka was the only country to incorporate all 25 categories of services we came across in the literature. South Africa and Rwanda had >75% of these categories at 19 and 20 categories, respectively. The arrangement of service categories in Vietnam was the least similar to those we encountered in the literature at only nine service categories (see Supplementary Appendix 3). However, we note that some of these categories are housed within bigger categories as demonstrated in the South Africa, Rwanda, and Sri Lanka columns of Supplementary Appendix 3.

When we examined the scope of services within the maternal health services categories, the most comprehensive list was observed within the South African policy literature with 27 specific recommendations. Every other country, with the exception of Burundi and Vietnam as stated earlier, included fewer than 20 specific service recommendations for FRH in the maternal health category. None of the countries we examined included the full scope of 34 service recommendations for maternal health found in academic and policy literature.

## Discussion

In this review, we highlight that, at the global level, the range of clinical service specifications that might be provided at FRHs, according to multiple, authoritative sources, are extensive but unevenly distributed across clinical areas. We identified 404 specific FRH service specifications and 15 broader statements on organizational arrangements were articulated. To our knowledge, this is the first report that has attempted to provide a contemporary account of expectations for FRH service delivery based on current literature.

We acknowledge several weaknesses in our research. First, our findings likely underestimate the scope of expected FRH services. Challenges in identifying literature on this topic required us to make pragmatic decisions to identify and focus on key information sources. In addition, many expectations may not be specified as they are felt to be implicit within a broader thematic area. For example, we found few recommendations in the arena of internal medicine, but the ability to provide ‘stroke care’ encompassing acute management to post-discharge rehabilitation might be implicit expectations of any FRH admitting adult patients. Secondly, we did not explore the potential impact of shaping FRH as delivery platforms in line with the existing (and unbalanced) global service expectations in the literature. We recognize that each country has likely selected service recommendations based on their needs and resources, as demonstrated by the country case studies, often reflecting a disconnect between the aspirations represented in the global literature and countries’ realities. Moreover, we did not examine the actual implementation of stated country-level service packages within their respective FRHs. Such studies were beyond the scope of this review. Third, findings from our country-level case studies are not intended to be generalizable but are used to illustrate the differences between countries and the aspirations presented in the literature review.

There is substantial variation in the number of specific service recommendations across service categories with recommendations for FRH representing traditional, broad clinical disciplines developed between the 1960s and 1980s, while progressive development of recommendations in some fields is lacking (WHO 1987, 1992). For pharmacy, an area that did not yield any recommendations in our search, guidance exists in WHO’s Essential Medicines List (EML) (WHO 2021a) and EML for children (WHO 2021b). However, these are not organized with regard to potential use at FRH or other service delivery levels. It is possible that our search strategies contributed to these apparent differences between clinical arenas, although we made efforts to identify recommendations from multiple professional association websites. Alternatively, differences may reflect historical priorities (e.g. maternal health), the emergence of vertical communicable disease programmes (e.g. HIV and TB), the prominent discourse around burden of disease, the earlier Millennium Development Goals (e.g. child and newborn health), and the attention given to ‘global health’ by different professional groups (e.g. the growth in interest in global surgery and laboratory services) (WHO 1987, 1992, Lopez and Murray 1998, Sachs and Mcarthur 2005, Mcarthur 2014). The emerging role of different professional groups, as advocates for essential services within their field of interest, is reflected in the themes covered by available *Lancet* Series and Commissions (Patel et al. 2018, Bukhman et al. 2020, Fleming et al. 2021). However, our findings could also represent a belief that some types of service do not belong at FRH rather than a lack of attention paid to some of these fields.

We did not examine the process through which FRH clinical service expectations were derived within our country’s case studies. Differences identified in both their categorization and content are expected, given country-level differences in health system organization and facility hierarchies (e.g. expected size of FRHs and the population they serve), population health needs, broad health system challenges faced, and resource availability (Mazhar et al. 2024). However, it is also conceivable that the absence of clear global guidance on a minimum set of expected clinical services at FRH contributes at least partially to country-level differences. In general, countries’ service specifications are not as extensive as those identified in our review of global recommendations, an observation noted by others (English and Opiyo 2011, English et al. 2017, Keene et al. 2019). A further aim of this paper is to highlight the growing potential tension between global service recommendations and countries’ ability to adopt such recommendations in their FRH. Many global recommendations are developed by disease or discipline-specific groups, yet countries face the challenge of integrating specific service recommendations into their primary and referral care systems and creating organizationally coherent FRH. Countries may make different decisions on the clinical services they aim to provide at the FRH or higher hospital levels based on their particular context. As countries navigate the various current recommendations, much might be learned by examining how countries make these decisions.

Our findings raise several questions. Is it important or helpful to define health service packages as an essential step in hospital planning (Watkins et al. 2017)? If so, in light of each country making context-specific health system planning decisions, how might countries navigate the increasing range of recommendations from the global health community in practice when making policy decisions? Is it appropriate to suggest a standardized minimum FRH service package appropriate for different population sizes being served that can be efficiently and effectively integrated to deliver safe, high-quality care? Our review demonstrated that most of the countries we reviewed had FRH policy suggestions that bear some resemblance to the 1992 WHO guidance for district hospitals. In each case study, we can assume FRHs were adapted to complement PHC objectives and manage the flow of referrals to higher levels of hospital care. However, it was beyond the scope of this study to explore how future priority-setting deliberations might be conducted and transparently documented. It is not clear how approaches such as a minimum list of essential services based largely on cost-effectiveness might be a helpful starting point for discussion. Although one aim might be to build up service scope rather than appear to remove and therefore ration services, it is not always clear how one moves from lists of cost-effective services to creating organizationally functional FRH (Watkins et al. 2017).

The authors of the recent *Lancet* Commission on PHC defined services that fall ‘beyond the scope of primary care’ rather than specifying where within the health system a service should be delivered (Hanson et al. 2022). FRH services could be assumed to fall within this category that is beyond the scope of primary care and at risk of neglect in health systems. The WHO has suggested countries identify ‘models of care’ aligned with essential health packages that encompass recommended roles for different levels of the health system including FRH (World Health Organization and the United Nations Children’s Fund (UNICEF) 2020). Thus, for each clinical service, the model may suggest how one level serves as a gateway to each successively higher level of care. Such an integrated approach is appealing but integration at one level, for example the FRH, may be challenging and decisions may be influenced by often powerful donor or medical professional voices potentially reflecting the imbalances seen in global recommendations. The gate-keeping function of ambulatory primary care may also be limited by ‘out of hours’ needs, when emergency services at FRH may be relied on to continue delivering services.

The complementarity of FRHs with wider primary care systems may be highly context dependent, undermining any effort at standardization across countries or proposed global service-specific recommendations (World Health Organization and the United Nations Children’s Fund (UNICEF) 2020). Previous efforts to standardize other aspects of the health system have proved challenging (Nabyonga-Orem et al. 2016), and historical attempts by the WHO Regional Office for Africa to standardize FRH services as part of the district health system were not always successful (WHO-AFRO 2013). Yet given FRH’s pivotal role both in support of primary care and as part of wider referral systems, defining a consensus on what services they should or should not provide at a country level seems important for equity considerations and UHC (Freijser et al. 2023). This will be influenced by the level of decentralization within each country and making such decisions will need to balance many competing demands. This paper does not advocate for a single approach but suggest that such decision-making processes and their effects are worthy of much greater research attention.

Our study provides pause for thought on how the global health community can strengthen recommendations on FRH as service delivery platforms and better leverage these important facilities’ potential to support UHC. Overall, our findings convey what may be a disconnect between the production of an extensive albeit patchwork set of global FRH clinical service specifications and the reality of FRH plans at the level of individual countries. These accumulating service expectations are not yet captured in broader FRH policy documents, and growing lists of recommendations made by experts in specific fields may not lend themselves to developing FRH holistically as physical or organizational entities. The development of the UHC Compendium may help prompt global and national discussion and transparent prioritization decisions on specific FRH service recommendations. However, countries will still be faced with challenging decisions on the roles and service delivery expectations of FRHs within their particular PHC and higher-level referral care systems (Mccord et al. 2015). The research community could support these efforts by considering some of the questions raised by these findings on FRH clinical roles, with due consideration to feasibility, complementarity, and optimization of service delivery scope and models of care (Anne 1990, Barnum and Kutzin 1993, Mcpake 1996, Rechel et al. 2016, Babalola et al. 2022, Freijser et al. 2023).

## Conclusion

In previous work, we have demonstrated that FRHs remain poorly defined as platforms of health care delivery and they appear to have fallen off the global health research and policy agendas in recent decades (Mazhar et al. 2024). Here, we note relatively incomplete specification of FRHs in terms of what they might provide as a holistic set of services. Yet careful thinking on the scope of FRH services in many countries is part of numerous other considerations of health system architecture spanning appropriate technologies, health professional training, and workforce planning among others. We believe these findings underscore the need for greater global attention to what is appropriate and feasible to deliver at the FRH level in different contexts. As countries make decisions on the organization of their health systems and the role of FRH, opportunities will exist to learn from these efforts to inform the development of efficient, high-quality FRH care in support of UHC.

## Supplementary Material

czaf021_Supp

## Reflexivity statement

This work was undertaken by a group of researchers based in both high- and low-resource countries across Africa, Asia, and Europe. The author group comprises four females and three males, with varying research seniority levels and academic backgrounds and qualifications. The lead researchers were two female junior researchers who drafted this paper with the guidance of Professor M.E., an Oxford-based researcher with decades of experience in East Africa. The authors from Burundi, Myanmar, Rwanda, and Nepal provided perspectives from countries with different health systems to those experienced by the lead researchers, which vastly strengthened the research and provided opportunities to consider how individual countries fit within a global picture.

## References

[R1] Anne M . The economics of hospitals in developing countries. Part II. Costs and sources of income. *Health Policy Plan* 1990;5:203–18. doi: 10.1093/heapol/5.3.203

[R2] Azen R, Walker CM. *Categorical Data Analysis for the Behavioral and Social Sciences: Chapter 2*. Routledge, 2021.

[R3] Babalola TK, Ojugbele HO, Shahwan M et al. Analysis of factors influencing technical efficiency of public district hospitals in Kwazulu-Natal province, South Africa. *Human Resour Health* 2022;20:80. doi: 10.1186/s12960-022-00777-2 PMC9682685 36419126

[R4] Barnum H, Kutzin J *Public Hospitals in Developing Countries: Resource Use, Cost, Financing*. Baltimore, 1993.

[R5] Bukhman G, Mocumbi AO, Atun R et al. The *Lancet* NCDI Poverty Commission: bridging a gap in universal health coverage for the poorest billion. *Lancet* 2020;396:991–1044. doi: 10.1016/S0140-6736(20)31907-3 32941823 PMC7489932

[R6] English M, Irimu G, Nyamai R et al. Developing guidelines in low-income and middle-income countries: lessons from Kenya. *Arch Dischildhood* 2017;102:846–51. doi: 10.1136/archdischild-2017-312629 PMC5564491 28584069

[R7] English M, Opiyo N. Getting to grips with grade—perspective from a low-income setting. *J Clin Epidemiol* 2011;64:708–10. doi: 10.1016/j.jclinepi.2010.07.016 21316192 PMC3319275

[R8] Fleming KA, Horton S, Wilson ML et al. The *Lancet* Commission on diagnostics: transforming access to diagnostics. *Lancet* 2021;398:1997–2050. doi: 10.1016/S0140-6736(21)00673-5 34626542 PMC8494468

[R9] Freijser L, Annear P, Tenneti N et al. The role of hospitals in strengthening primary health care in the Western Pacific. *Lancet Reg Health West Pac* 2023;33:100698. 10.1016/j.lanwpc.2023.100698 PMC9984548 36880058

[R10] Hanson K, Brikci N, Erlangga D et al. The *Lancet Global Health* Commission on financing primary health care: putting people at the centre. *Lancet Glob Health* 2022;10:E715–72. doi: 10.1016/S2214-109X(22)00005-5 35390342 PMC9005653

[R11] Keene CM, Aluvaala J, Murphy GAV et al. Developing recommendations for neonatal inpatient care service categories: reflections from the research, policy and practice interface in Kenya. *BMJ Global Health* 2019;4:E001195. doi: 10.1136/bmjgh-2018-001195 PMC6441269 30997163

[R12] Lopez AD, Murray CCJL. The global burden of disease, 1990–2020. *Nat Med* 1998;4:1241–43. doi: 10.1038/3218 9809543

[R13] Mazhar R, Willows T, Bhattarai S et al. First level, first referral, or district hospital? Primary or secondary care facilities? Clearer thinking is needed to inform global health research and policy. *Health Policy Plan* 2024;39:224–32. doi: 10.1093/heapol/czad120 38386923 PMC11031140

[R14] Mcarthur JW . The origins of the millennium development goals. *SAIS Rev* 2014;34:5–24.

[R15] Mccord C Kruk ME Mock CN et al. Organization of essential services and the role of first-level hospitals. Vol. 1. In: Jamison DT, Nugent R, Gelban H et al. (eds.), *Disease Control Priorities*. New York: World Bank, 2015, 213–31. 26741000

[R16] Mcpake BI . Public autonomous hospitals in sub-Saharan Africa: trends and issues. *Health Policy* 1996;35:155–77. doi: 10.1016/0168-8510(95)00778-4 10156652

[R17] Nabyonga-Orem J, Tumusiime P, Nyoni J et al. Harmonisation and standardisation of health sector and programme reviews and evaluations—how can they better inform health policy dialogue? *Health Res Policy Syst* 2016;14:1–8. doi: 10.1186/s12961-016-0161-9 27986084 PMC5162096

[R18] Patel V, Saxena S, Lund C et al. The *Lancet* Commission on global mental health and sustainable development. *Lancet* 2018;392:1553–98. 30314863 10.1016/S0140-6736(18)31612-X

[R19] Rechel B, Džakula A, Duran A et al. Hospitals in rural or remote areas: an exploratory review of policies in 8 high-income countries. *Health Policy* 2016;120:758–69. doi: 10.1016/j.healthpol.2016.05.011 27312144

[R20] Sachs J, Mcarthur J. The Millennium Project: a plan for meeting the Millennium Development Goals. *Lancet* 2005;365:347–53. doi: 10.1016/S0140-6736(05)17791-5 15664232

[R21] UNFPA . 2023. *Publications* [Online]. UNFPA. https://www.unfpa.org/publications (14 June 2023, date last accessed).

[R22] Van Zyl C, Badenhorst M, Hanekom S et al. Unravelling ‘low-resource settings’: a systematic scoping review with qualitative content analysis. *BMJ Global Health* 2021;6:e005190. doi: 10.1136/bmjgh-2021-005190 PMC8183220 34083239

[R23] Watkins DA Jamison DT Mills T et al. Universal health coverage and essential packages of care. In: Jamison DT, Gelband H and Horton S et al. (eds), *Disease Control Priorities: Improving Health And Reducing Poverty*. Washington, DC: The International Bank for Reconstruction and Development/The World Bank, 2017, e11–14.

[R24] WHO . *Declaration of Alma-Ata*. World Health Organization. Regional Office for Europe, 1978.

[R25] WHO . Hospitals and health for all. Report of the WHO Expert Committee on the role of hospitals at the first referral level. *World Health Organ Tech Rep Ser* 1987;744:1–82. 3107221

[R26] WHO . *The Hospital in Rural and Urban Districts. Report of a WHO Study Group on the Functions of Hospitals at the First Referral Level*. Geneva: World Health Organization (WHO), 1992. 1585664

[R27] WHO . *District Hospitals: Guideline for Development* [Online]. WHO Regional Office for the Western Pacific, 1996.

[R28] WHO . 2021a. *WHO Model List of Essential Medicines* [Online]. 2021. https://www.who.int/publications/i/item/WHO-MHP-HPS-EML-2021.02 (6 July 2023, date last accessed).

[R29] WHO . 2021b. *WHO Model List of Essential Medicines for Children—8th List* [Online]. https://www.who.int/publications/i/item/WHO-MHP-HPS-EML-2021.03 (6 July 2023, date last accessed).

[R30] WHO . *UHC Compendium: Health Interventions for Universal Health Coverage* [Online]. WHO. https://www.who.int/universal-health-coverage/compendium#:~:text=The%20UHC%20Compendium%20is%20a,health%20services%20and%20health%20interventions. (2 July 2023, date last accessed).

[R31] WHO . 2023a. *Health Topics* [Online]. https://www.who.int/health-topics (12 August 2023, date last accessed).

[R32] WHO-AFRO. District health in Africa: progress and prospects 25 years after the Harare Declaration. In: WHO-AFRO, ed. Regional Conference on Health District, Senegal, 2013, 2–6.

[R33] World Health Organization and the United Nations Children’s Fund (UNICEF) . *Operational Framework for Primary Health Care: Transforming Vision into Action*. Geneva: World Health Organization and the United Nations Children’s Fund (UNICEF), 2020.

