# Supplementary material for: First referral hospitals in low-resource settings: a narrative review of expectations for clinical service provision
Source: Health Policy Plan. 2025 Mar 31;40(6):652–60. doi: 10.1093/heapol/czaf021 (PMC12160796; doi:10.1093/heapol/czaf021)
Supplement: czaf021_Supp [file czaf021_supp.zip › suppl_data/FRH_Services Paper_Supplementary Material.docx]

**Supporting material: Table of contents**

| Appendix 1: Academic literature search strategy……………………………………………………………... |  |
| --- | --- |
| Appendix 2: Combined list of FRH clinical services from academic and policy literature……………………. |  |
| Appendix 3: Comparison of country case studies’ classification of FRH expected services..……...…………. |  |
| Appendix 4: Comparison of country case studies’ FRH expected maternal health services…………………... |  |
| Supplementary Material 1: Case study data extraction template……………………………………………… |  |
| Supplementary Material 2: Academic articles included for data extraction….……………………………….. |  |
| Supplementary Material 3: Policy documents included for data extraction………………………………… |  |

**Appendix 1: Academic literature search strategy**

| Inclusion criteria:   - Provide recommendations of clinical services expected to be delivered by FRH (or an equivalent synonym) - That are global or LMIC region focused   Exclusion criteria:   - Provided health service recommendations without specifying the role of FRH (or an equivalent synonym) - Clinical service recommendations that refer generally to hospitals without mentioning a specific level. - Specifically address high income settings, or - Are country-focused, or provided recommendations that fall outside of the scope of health services, such as the positioning, financing, resourcing or management of FRH or their roles and functions within the broader health system such as outreach activities, data capturing, training for health care workers etc. |
| --- |

Step 1: Structured literature review

Articles excluded (n=641)

Articles title and abstract /summary screened from search strategy (n=680)

**Screening**

Articles excluded in Round 1 (n= 34)

**Full text review-**

**Round 1**

Articles from search strategy assessed for eligibility through full text review (n =39)

Articles from pre-existing repository assessed for eligibility through full text review (n=16)

Articles excluded in Round 1 (n= 13)

Articles excluded in Round 2 (n= 4)

**Full text review-**

**Round 2**

Articles from search strategy re-assessed for inclusion in second review (n = 5)

Articles from pre-existing repository re-assessed for inclusion in second review (n=3)

Articles excluded in Round 2 (n= 1)

Articles included in final review from search strategy (n=1)

Articles included in final review from pre-existing repository (n=2)

(n = 3)

Step 2: Review of The Lancet Commissions

Articles excluded

(n= 54)

Articles title and abstract /summary screened (n=85)

**Screening**

Articles excluded in Round 1 (n=6)

Articles assessed for eligibility through full text review (n = 31)

**Full text review- Round 1**

Articles excluded in Round 2 (n=20)

Articles re-assessed for inclusion in second review (n = 25)

**Full text review-**

**Round 2**

Articles included in final review

(n = 5)

Step 3: Review of The Lancet Series

Articles excluded

(n= 1876)

Articles title and abstract /summary screened (n=1922)

**Screening**

**Full text review- Round 1**

Articles excluded in Round 1 (n=28)

Articles assessed for eligibility through full text review (n = 46)

**Full text review-**

**Round 2**

Articles excluded in Round 2 (n=10)

Articles re-assessed for inclusion in second review (n = 18)

Articles included in final review

(n = 8)

**Appendix 2: Combined list of FRH clinical services from academic and policy literature**

**Service specification = 404**

| Emergency, trauma and acute care (n=87) |
| --- |
| **Organisational arrangements**  In addition to the specific service specifications listed below, the presence of a specific emergency area or unit is specified, with a core of fixed/non-rotating staff with capacity to triage (assessment of emergency and priority signs) as well as access to operative care for injuries, 24-hour access to surgical care, anaesthesia, critical care services and emergency obstetric and surgical care; and capacity to transport and refer the severely ill patient to a higher level of care. The importance of having mass casualty management plans and emergency incident command systems for all hospitals is also specified.  **Service specifications**  **Specific capability for care of the severely ill adult**   1. Management of haemorrhagic shock 2. Management of hypovolaemic shock 3. Management of anaphylactic shock 4. Management of cardiogenic shock 5. Management of septic shock 6. Initial emergency management for all severely ill patients with difficulty breathing and specifically:    1. Management respiratory distress in patients with suspected severe pneumonia or acute lung injury and without shock    2. Management of patients with severe respiratory distress from acute bronchospasm (from either asthma or chronic obstructive pulmonary disease or other causes of acute wheezing)    3. Management if patients with severe respiratory distress from acute pulmonary oedema or fluid overload 7. Management of acute decompensated cardiac problems 8. Management of delirium 9. Management of diabetic ketoacidosis 10. Management of hypoglycaemia 11. Management of steroid deficiency (Addison’s disease; adrenal insufficiency) 12. Management of intoxication or overdose, or withdrawal from injecting or other use of opioids, amphetamine-type stimulants or cocaine 13. Management of acute alcohol withdrawal and intoxication 14. Management of poisoning (spanning poisons relevant to adults and children):     1. Ingested poisons or overdose of medicines     2. Inhaled poisons     3. Chemicals on the skin or in the eye 15. Snakebite assessment and treatment in adults 16. Burn management including assessing and classifying severity and initial stabilization   **Trauma and injuries in children**   1. Primary survey or initial assessment 2. Secondary survey   **Trauma: for the acutely injured patient (all ages)**   1. Assessment of and treatment for immediately life-threatening injuries 2. Resuscitation and stabilization 3. Wounds (soft tissue injuries) 4. Administration of oxygen therapy for trauma patients 5. Immobilization of the spine 6. Management of serious head injury 7. Management of tension pneumothorax or massive haemothorax 8. Treatment for sucking chest wound 9. Application of pressure to stop bleeding 10. Application of pelvic binder 11. Violence and injury prevention 12. Management of rape or abuse in adolescents and adults   **Therapeutic procedures (all ages)**   1. Urinary catheter insertion – female 2. Urinary catheter insertion – male 3. Suprapubic catheter 4. Reduction of paraphimosis 5. Marsupialization for Bartholin’s cyst or abscess 6. Inserting a nasogastric (NG) tube 7. Gastric lavage 8. Venous cutdown 9. Lumbar puncture 10. Thoracentesis (chest tap) 11. Chest tube (intercostal chest drain) 12. Paracentesis (abdominal tap) 13. Arthrocentesis (joint aspiration) 14. Pericardiocentesis   **Other emergency care in adults**   1. Management of heavy upper gastrointestinal bleeding 2. Management of large nose bleed (epistaxis) 3. Application of aortic compression   **Emergency management specified for children:**   1. Management of choking in the infant / child 2. Drowning 3. Electrocution 4. Treatment for severe dehydration in an emergency setting in children 5. Emergency treatment for a child with severe malnutrition 6. Management of child presenting with an airway or severe breathing problem 7. Management of child presenting with shock 8. Management of child presenting with lethargy, unconsciousness or convulsions   **Management of specific poisoning in children including:**   1. Corrosive compounds 2. Petroleum compounds 3. Organophosphorus and carbamate compounds 4. Paracetamol 5. Aspirin and other salicylates 6. Iron 7. Morphine and other opiates 8. Carbon monoxide 9. Prevention of poisoning   **Management of common causes of envenoming in children**   1. Snake bite 2. Scorpion sting 3. Other sources of envenoming   **Advanced airway management (for district clinicians with training):**   1. Tracheal intubation 2. Confirmation of endotracheal tube (ETT) placement 3. Post-intubation care 4. Ventilation for intubated patient 5. Sedation for intubated patient 6. Monitoring of intubated patients 7. Manual ventilation (bagging) 8. Cricothyroidotomy for life threatening upper airway obstruction   **Emergency – medical interventions**   1. Provision of sequential bronchodilators for wheezing 2. Insertion of IV / IO and administration of fluids rapidly 3. Administration of naloxone 4. Administration of glucose 5. Administration of diazepam IV or rectally 6. Placement of patient in recovery position 7. Administration of empirical IV/IM antibiotics for emergency management 8. Administration of emergency antimalarial treatment if falciparum malaria is possible 9. Administration of emergency antiviral treatment 10. Administration of ketamine 11. Management of the violent or very agitated patient 12. Management of the suicidal/self-harm patient |

| Advanced medical care (including communicable and non-communicable diseases) (n=7) |
| --- |
| **Organisational arrangements**  In addition to the specific service specifications listed below, the presence of emergency or high dependency units to address acute manifestations of chronic conditions is specified, along with a commitment to integration of pain treatment into service delivery district hospitals and to ensuring adequate geographical coverage to make pain relief accessible to all who need it across the country.  **Service specifications**   1. Resuscitation with basic life support measures 2. Resuscitation with advanced life support measures, including surgical airway 3. Provision of aspirin for all cases of suspected acute myocardial infarction 4. Medical management of acute, decompensated heart failure 5. Management of advanced malignancies and other end-stage NCDs with pain (i.e., using tamoxifen or imatinib, morphine etc) 6. Management of treatment failure for Yaws 7. Identification and management of adverse events from Yellow Fever vaccine |

| Ear Nose and Throat (n=3) |
| --- |
| **Service specifications**   1. Management of mastoiditis in children 2. Management of acute otitis media in children 3. Management of chronic otitis media in children |

| Eye health (n=0) |
| --- |
| **Organisational arrangements**  The presence of an ophthalmic outpatient team is specified. No specific eye health services were identified. |

| Surgery & Anaesthesia (n=83) |
| --- |
| **Organisational arrangements**  In addition to the specific service specifications listed below, the presence processes in place for postoperative recovery care are specified. Emphasis is also placed on building cancer surgical into FRH.  **Service specifications**   1. Laparotomy / general surgery for acute abdominal conditions 2. Caesarean delivery 3. Treatment of open fracture 4. Wound debridement 5. Dilation and curettage 6. Closed fracture reduction 7. Hernia repair 8. Contracture release 9. Superficial soft tissue tumour resection 10. Gastroscopy 11. Cholecystectomy 12. Intracranial haematoma evacuation 13. Thyroidectomy 14. Anal fissures and fistula 15. Management of acute critical limb ischaemia with amputation as a last resort 16. Relief of urinary obstruction by catheterisation or suprapubic cystostomy 17. Appendectomy 18. Hernia repair, including emergency surgery 19. Colostomy 20. Basic skin grafting 21. Management of bowel obstruction 22. Repair of perforations (e.g., perforated peptic ulcer and typhoid ileal perforation) 23. Management of osteomyelitis, including surgical 24. Escharotomy or vasciotomy debridement for refractory cases 25. Fracture reduction; Irrigation and debridement of open fractures; Placement of external fixator and use of traction for fractures 26. Tube thoracostomy 27. Trauma laparotomy 28. Management of tracheostomy 29. Trauma-related amputations 30. Surgery to manage buruli ulcer i.e., excision of nodules, papules, plaques and ulcers 31. Surgical and anaesthesia care for lymphatic filariasis patients 32. Feeding gastrostomy 33. Management of head injuries 34. Circumcision 35. Craniotomy 36. Plaster techniques 37. Extraction of teeth 38. Intussusception 39. Ruptured vagina 40. Ruptured uterus 41. Ruptured cervix 42. Perforated intestine 43. Ulcers 44. Sterilization- female 45. Sterilization- male 46. Symphysiotomy 47. Control of epistaxis 48. Version and extraction 49. Hydrocelectomy 50. Removal of foreign body 51. Incision and drainage of abscesses 52. Bowel obstruction in children 53. Abdominal wall defects in children 54. Myelomeningocele in children 55. Congenital dislocation of the hip in children 56. Talipes equinovarus (club foot) in children   **Procedures potentially needing visiting specialist support:**   1. Thoracic surgery 2. Transurethral resection of prostate 3. Uretero-renoscopy 4. Vesicovaginal fistula 5. Basic skin flaps 6. Rectal prolapse repair 7. Cataract surgery 8. Cleft lip and palate repair   **Injuries in children:**   1. Burns in children 2. Head injuries in children 3. Chest injuries in children 4. Abdominal injuries in children 5. Fractures in children 6. Principles of wound care in children   **Abdominal problems in children:**   1. Abdominal pain in children 2. Bowel obstruction after the neonatal period in children 3. Testicular torsion in children 4. Rectal prolapse in children   **Infections requiring surgery in children:**   1. Abscess in children 2. Septic arthritis in children 3. Pyomyositis in children   **Anaesthesia:**   1. Care of airways 2. Conduction anaesthesia 3. Anaesthesia for emergency cases 4. Intubation and management of its complications 5. Postoperative recovery care for adults and children 6. Induction and maintenance of general anaesthesia |

| Female reproductive health (n=13) |
| --- |
| **Organisational arrangements**  In addition to the specific service specifications listed below, the presence of processes to offer counselling and provision of the full range of family planning methods, screening for cancer of the cervix and of the breast and to offer identification of initial needs of the infertile couple and referral (including HIV discordant couples) are specified.  **Service specifications**   1. Management of tubal ligation/vasectomy/insertion and removal of implants, difficult removal of devices etc. 2. Uterine evacuation for first-trimester and incomplete abortions 3. Diagnosis and treatment of common complications of abortion including infection, bleeding or injury 4. Management of ectopic pregnancy 5. Cervical cancer screening: pap smear 6. Cervical cancer screening: visual screening 7. Cervical biopsy and endocervical curettage 8. Cervical colposcopy 9. Clinical breast examination 10. Endometrial biopsy 11. Human papillomavirus DNA testing and treatment of precancerous lesions 12. Intrauterine device (IUD) placement 13. Prevention and management of STIs |

| Maternal health (n=34) |
| --- |
| **Organisational arrangements**  In addition to the specific service specifications listed below, the presence of essential equipment such as clean birth kits and processes to provide antenatal care, 24-hour acute care services, normal delivery care (including companion of choice to support the woman during delivery) with clean birth practices, comprehensive emergency obstetric and newborn care (mandatory for obstetric units to keep stored blood available, especially type O negative blood and fresh frozen plasma), post-natal care (woman and well newborn), and care of the sick newborn are specified, with functional and well organised referral mechanisms. The presence of a maternal death review committee is recommended.  **Service specifications**  **Antenatal**   1. Antenatal care 2. Treatment of medical conditions in pregnancy, side effects and/or complications. 3. Antimalarial Intermittent preventive treatment (IPT) and promotion of insecticide treated nets (ITN) 4. Deworming 5. Assessment of female genital mutilation 6. Treatment of mild to moderate opportunistic infections 7. Treatment of simple and complicated malaria cases in pregnancy 8. Monitoring and assessment of maternal well-being, prevention and detection of complications (e.g. hypertension, infections, bleeding, anaemia) 9. Vitamin A administration for mother 10. Management of vaginal bleeding in early pregnancy, late pregnancy and during labour 11. Administration of magnesium sulphate / manage eclampsia or pre-eclampsia   **Labour and delivery**   1. Pain management strategy for surgical abortions 2. Delivery and immediate care of the newborn baby 3. Caesarean section 4. Safe blood transfusion 5. Induction and augmentation of labour 6. Monitoring progress of labour, maternal and foetal well-being with partograph 7. Infection prevention during labour 8. Supportive care and pain relief during labour 9. Detection of problems and treatment of complications of labour (e.g. malpresentations, prolonged and/or obstructed labour, hypertension, bleeding, and infection) 10. Treatment of abnormalities and complications (e.g. prolonged labour, vacuum extraction; breech presentation, episiotomy, repair of genital tears, manual removal of placenta) 11. Management of serious complications (e.g. obstructed labour, foetal distress, preterm labour, severe peri- and postpartum haemorrhage) 12. Emergency hysterectomy   **Postnatal**   1. Initiation of breastfeeding 2. Immediate postpartum care of mother 3. Support for the family if maternal or perinatal death 4. Counselling for family planning including insertion of IUDs 5. Management of vaginal bleeding postpartum 6. Massaging uterus and expelling clots 7. Condom inflation over foley catheter to tamponade uterine bleeding 8. Application of bimanual uterine compression 9. Administration of oxytocin 10. Manual removal of the placenta if postpartum bleeding 11. Administration of misoprostol for postpartum bleeding if no response to oxytocin plus ergometrine |

| Newborn health (n=25) |
| --- |
| **Organisational arrangements**  In addition to the specific service specifications listed below, the support, revitalization, expansion and institutionalization of the Baby-Friendly Hospital Initiative is specified including promotion of rooming in and healthy behaviours (e.g., hygiene,, warmth),with a particular emphasis on the promotion, protection and support for exclusive breastfeeding and support for safer infant feeding options. Kangaroo Mother Care is specified, along with monitoring and assessment of well-being and response to maternal concerns for early detection of maternal / neonatal complications or emerging conditions, and mechanisms in place for referral as needed. The presence of newborn screening units to identify newborns with life-threatening congenital conditions such as sickle cell disease are also specified, along with neonatal drug formulations and prescribing guidance, and post-discharge follow-up for at-risk babies. It is recommended that every district, or equivalent subnational planning unit, should have a facility providing level-2 Small and Sick Newborn Care (SSNC).  **Service specifications**   1. Eye infection prophylaxis 2. Immunization 3. Treatment of local infections (skin, cord, eye, mouth) 4. Identification, initial management and referral of a newborn with injury, malformation or birth defects 5. Management of preterm babies with breathing problem or unable to feed orally 6. Resuscitation 7. Management of low or very-low birth weight babies 8. Management of neonatal sepsis 9. Management of meningitis 10. Management of severe birth asphyxia / encephalopathy 11. Management of severe jaundice 12. Management of prematurity 13. Management of HIV exposure 14. Presumptive treatment of congenital syphilis   **Supportive care**   1. Comfort and pain management 2. Temperature / thermal control 3. Assisted feeding for optimal nutrition (cup feeding and nasogastric feeding) 4. Safe administration of oxygen 5. Continuous Positive Airway Pressure (CPAP) 6. Prevention of apnoea 7. Detection and management of hypoglycaemia 8. Detection and management of anaemia 9. Seizure management 10. Safe administration of intravenous fluids 11. Cessation of resuscitation |

| Child health (n=44) |
| --- |
| **Organisational arrangements**  In addition to the specific service specifications, the presence of toys and play area and therapy for children in hospitals is specified.  **Service specifications**  **Supportive care**   1. Maintenance fluid management 2. Oxygen therapy 3. Administration of IV fluids for shock in a child without severe acute malnutrition 4. Administration of IV fluids for shock in a child with severe acute malnutrition 5. Pain control 6. Medical management of anaemia   **Transfusion**   1. Giving blood transfusion 2. Identifying and managing transfusion reactions   **Assessment and management of pneumonia and complications:**   1. Assessment of child presenting with cough for pneumonia including severe pneumonia 2. Pleural effusion and empyema 3. Lung abscess 4. Pneumothorax   **Management of the following conditions presenting with wheeze:**   1. Bronchiolitis 2. Asthma 3. Wheeze with cough or cold   **Management of the following conditions presenting with stridor:**   1. Viral croup 2. Diphtheria 3. Epiglottitis 4. Anaphylaxis   **Management of the following conditions presenting with chronic cough:**   1. Pertussis 2. Tuberculosis 3. Foreign body inhalation 4. Heart failure 5. Rheumatic heart disease   **Management of child presenting with fever**   1. Fever lasting 7 days or less 2. Fever lasting longer than 7 days   **Malaria management**   1. Severe malaria 2. Uncomplicated malaria   **Meningitis management**   1. Bacterial meningitis 2. Meningococcal epidemics 3. Tuberculous meningitis 4. Cryptococcal meningitis   **Measles management**   1. Severe complicated measles 2. Non-severe measles   **Management of other severe infections**   1. Septicaemia 2. Typhoid fever 3. Urinary tract infection 4. Septic arthritis or osteomyelitis 5. Dengue including Severe dengue 6. Rheumatic fever   **Management of acute diarrhoea**   1. Severe dehydration, Some dehydration, No dehydration 2. Dysentery   **Management of persistent diarrhoea:**   1. Severe persistent diarrhoea 2. Persistent diarrhoea (non-severe) |

| Adolescent health (n=0) |
| --- |
| **Organisational arrangements**  No specific services identified, however the provision of adolescent hospital care (e.g. for HIV and other chronic diseases) is specified. |

| Mental health (n=1) |
| --- |
| **Organisational arrangements**  In addition to the specific service specifications listed below, the presence of specialist mental health staff if specified (the composition will vary depending resources, and preferably should consist of a multidisciplinary team, but at least a prescribing clinician), as well as a severe mental health outpatient team. Other organisational arrangements are disaggregated by low, medium and high resource settings. While the presence of outpatient clinics, supported by acute inpatient care in general hospitals and referral pathways to tertiary care are specified in all settings, in medium resource settings these are complemented by integration of mental health care with other secondary health care (e.g., maternal and child health, HIV) while in high resource settings these are complemented by availability of a full range of evidence-based psychosocial interventions delivered by trained experts and a full range of evidence-based pharmacological interventions available.  **Service specifications**   1. Management of schizophrenia using generic anti-psychotic medications and psychosocial treatment |

| Non-communicable diseases and cancer (n=22) |
| --- |
| **Organisational arrangements**  In addition to the specific service specifications listed below, the institutionalisation of smoke-free policies in hospitals are specified.  **Service specifications**  **NCD related**   1. Severe anaemia management 2. Provision of inpatient smoking cessation care (in the form of brief advice and provision of NRT in preoperative and postoperative units) as well as outpatient community cessation care 3. Management of acute exacerbations of asthma and COPD 4. Management of chronic pulmonary diseases 5. Severe NCD outpatient teams: management of type 1 diabetes 6. Management of Type 2 diabetes 7. Medical management of chronic heart failure 8. Relief of refractory suffering and acute pain related to cirrhosis and advanced malignancies, heart failure, and kidney disease. 9. Combination therapy for individuals with moderate to severe rheumatoid arthritis   **Cancer related**   1. Chemotherapy - High-risk acute lymphoblastic leukaemia 2. Chemotherapy - High-stage Burkitt lymphoma and Hodgkin lymphoma 3. Standard treatment (limited simple chemotherapy less complex surgery) for low-stage solid tumours 4. Palliative or survivorship care 5. Early identification of breast and cervical cancer: Fine needle aspiration (Breast) (FNA) 6. Early identification of breast and cervical cancer: Cervical cancer screening: Pap smear 7. Early identification of breast and cervical cancer: Cervical cancer screening: visual screening 8. Early identification of breast and cervical cancer: Colposcopy, cervical biopsy and endocervical curettage 9. Endocervical curettage (ECC) as diagnostic tests for cervical pre-cancer 10. Loop electrosurgical excision procedure (LEEP) 11. Skin biopsy – shaving or scraping, punch and excision 12. Lymph node biopsy (excisional) 13. Bone marrow aspiration and biopsy |

| Communicable diseases) (n=4) |
| --- |
| **Service specifications**   1. Diagnosis and treatment for category 1,2 and 3 buruli ulcers with antibiotics and reduction/prevention of recurrence 2. Chikungunya management: physician evaluation and hydration therapy 3. Clinical management of inpatient malaria cases 4. Management and surveillance of vaccine preventable diseases |

| TB/HIV (n=25) |
| --- |
| **Organisational arrangements**  In addition to the specific service specifications listed below, the presence of processes to offer routine HIV testing and counselling, care for HIV positive men and women, and prevention of mother to child transmission of HIV (PMTCT) (mode of delivery advice, early infant diagnosis in HIV exposed children and infant feeding counselling) are specified in addition to availability of treatment of severe HIV infection and ARV regimens for PMTCT including ART. In addition, presence of process for discharge from hospital and clinical follow up planning and/or referral as required are specified.  **Service specifications**  **Childhood TB**   1. Lumbar puncture and pleural taps 2. Read CXRs 3. Manage common side-effects and more serious cases of disease (e.g. miliary TB) 4. Tuberculin skin tests (TSTs) 5. Initiation and maintenance of TB therapy   **Childhood HIV**   1. Clinical diagnosis 2. HIV counselling 3. Testing and diagnosis of HIV infection 4. Clinical staging 5. Antiretroviral therapy for HIV- exposed & HIV-infected children and cotrimoxazole prophylaxis 6. Side-effects and monitoring 7. When to change treatment 8. Promotion of vaccination 9. Co-trimoxazole prophylaxis 10. Nutrition 11. Management of HIV-related Tuberculosis 12. Management of HIV-related Pneumocystis jiroveci pneumonia 13. Management of HIV-related Lymphoid interstitial pneumonitis 14. Management of HIV-related Fungal infections 15. Management of HIV-related Kaposi sarcoma 16. Palliative and end-of-life care: Pain control 17. Palliative and end-of-life care: Management of anorexia, nausea and vomiting 18. Palliative and end-of-life care: Prevention and treatment of pressure sores 19. Palliative and end-of-life care: Care of the mouth 20. Palliative and end-of-life care: Psychosocial support |

| Nutrition and malnutrition (n=14) |
| --- |
| **Organisational arrangements**  In addition to the specific service specifications listed below, the presence of processes in place to offer nutrition counselling for children, supporting breastfeeding , offering nutritional management of sick children and offering discharge to outpatient nutritional care are specified.  **Service specifications**  **Management of (severe) malnutrition in children and infants**   1. Hypoglycaemia 2. Hypothermia 3. Dehydration 4. Electrolyte imbalance 5. Infection 6. Micronutrient deficiencies 7. Initial re-feeding 8. Catch-up growth feeding 9. Sensory stimulation 10. Eye problems 11. Severe anaemia 12. Skin lesions in kwashiorkor 13. Continuing diarrhoea 14. Tuberculosis |

| Gender Based Violence (n=2) |
| --- |
| **Organisational arrangements**  In addition to the specific service specifications listed below, the presence of processes to ensure abortion services are accessible where it is not against the law, as well as protocols for evidence collection in cases of suspected rape and availability of 'rape kits' are specified. The role of all facilities in caring for and supporting survivors of GBV is specified, and upholding their right to the best possible health-care available at every level of health-care delivery integrated into existing health services as much as possible rather than offered only as stand-alone services.  **Service specifications**   1. Screening for and management of signs/symptoms of domestic violence and sexual assault 2. Clinical management of sexual violence survivors |

| Laboratories (n=33) |
| --- |
| **Organisational arrangements**  In addition to the specific service specifications listed below, diligence in obtaining samples; timely communication for sample processing; efficient transfer of material; prompt reporting of results; a reliable system of archiving and storage of samples; and broader system support and basic working equipment for analysis and processing are specified, with uninterrupted supply chain of quality reagents, equipment procurement, and maintenance. Recommendations are made for ‘tier-two laboratories’ at district-level hospitals as part of an integrated network of tiered laboratories with an appropriate ratio of laboratory technicians, technologists, and assistants to pathologists and clinical laboratory scientists.  **Service specifications**  **Haematology & transfusion**   1. Full blood count with differential 2. Erythrocyte sedimentation rate (ESR) 3. Type and cross match for transfusion 4. Fresh Frozen Plasma (obstetric emergencies) 5. Crude clotting time   **Chemistry**   1. Serum electrolytes 2. Amylase 3. Blood sugar (glucose) 4. Serum creatinine and blood urea nitrogen (BUN) 5. Serum alanine aminotransferase (ALT) 6. Bilirubin determination for neonates 7. Lactic acid 8. Chemistry (protein, glucose etc) for cerebrospinal fluid (CSF), urine, thoracentesis, and paracentesis 9. Blood gas 10. HbA1c 11. Cardiac markers 12. Faecal Immunochemical Test 13. Faecal occult blood   **Basic microscopy, microbiology and parasitology**   1. Basic microscopy for cerebrospinal fluid (CSF), urine, thoracentesis, and paracentesis 2. Stool microscopy for ova and parasites 3. Malaria thin and thick blood films or RDT 4. Taking stool samples, including Cary-Blair for cholera 5. Gram stain 6. Saline and potassium hydroxide (KOH) wet mounts (for bacterial vaginosis (BV) or trichomonas) 7. Blood and sputum cultures (may be sent out) 8. Acid fast bacilli (Ziehl-Neelsen) smear microscopy   **Diagnostic assays**   1. Rapid HIV antibody tests (first, second and third tests) 2. CD4 absolute count and percentage 3. Cryptococcal antigen (CrAg- serum or CSF) or India ink stain of CSF 4. Hepatitis B enzyme immunoassay (EIA) 5. Syphilis – rapid plasma reagin (RPR) 6. WHO-approved molecular testing such as Xpert MTB/RIF 7. Qualitative HIV virological (RNA, DNA, or US p24 Ag, Lymphocyte CD4*) 8. Immunoassay / serology    1. Chikungunya    2. Dengue fever    3. HBV    4. HCV    5. Yellow fever    6. Buruli ulcer assay Lab-IA (EIA/ECL/RDT) |

| Blood bank services (n=0) |
| --- |
| **Organisational arrangements**  The presence of adequate supplies of safe blood is specified, in addition to storage of blood for blood transfusion for children. |

| Radiology / Imaging / Electrophysiology (n=4) |
| --- |
| **Service specifications**   1. Electrocardiogram 2. Chest radiography 3. Ultrasonography for cancer diagnosis 4. Breast ultrasound and biopsy |

| Palliative care (n=3) |
| --- |
| **Organisational arrangements**  The presence of one or more doctors and nurses with at least basic palliative care training and a social worker who provides inpatient and outpatient palliative care as one official responsibility is specified.  **Service specifications**   1. Outpatient palliative care for moderate to severe symptoms of cervical cancer, or control of refractory suffering and ongoing care for patients with well controlled symptoms 2. Inpatient palliative care for moderate to severe symptoms of cervical cancer, or control of refractory suffering and ongoing care for patients with well controlled symptoms 3. Palliative care for TB |

| Rehabilitation (n=0) |
| --- |
| **Organisational arrangements**  Capacity for diagnosis and treatment of disability is specified, as well as presence of specialized rehabilitation units for inpatients with complex needs. |

Appendix 3: Comparison of country case studies’ classification of FRH expected services ‘-‘ = data not included in documents reviewed

**Appendix 4: Comparison of country case studies’ FRH expected maternal health services**

**Supplementary Material 1: Case study MS Word template**

| Country |  |
| --- | --- |
| Population size |  |
| Income group |  |
| Hospital beds/population |  |
| Administrative structure |  |
| Brief background |  |
| Healthcare administration |  |
| Financing of hospital care |  |
| Levels and types of hospitals |  |
| Diagram of hospital care hierarchy |  |
| Key timeline of policy changes |  |
| Identified issues affecting hospital care |  |
| Does primary care concept include hospital care? |  |
| Contextual first referral level hospital |  |
| Defined minimum service package and staffing norms |  |

**Supplementary Material 2: Academic articles included for data extraction**

| Source | Document | Year published | Link |
| --- | --- | --- | --- |
| Academic publication (from structured search) | District health systems in a neoliberal world: a review of five key policy areas | 2003 | <https://pubmed.ncbi.nlm.nih.gov/14661938/> |
| The Lancet Series | Scale up of services for mental health in low-income and middle-income countries | 2011 | [https://www.theLancet.com/journals/Lancet/article/PIIS0140-6736(11)60891-X/fulltext](https://www.thelancet.com/journals/lancet/article/PIIS0140-6736(11)60891-X/fulltext) |
| The Lancet Series | Improvement of pathology in sub-Saharan Africa | 2013 | [https://www.theLancet.com/journals/Lancet/article/PIIS1470-2045(12)70598-3/fulltext](https://www.thelancet.com/journals/lancet/article/PIIS1470-2045(12)70598-3/fulltext) |
| The Lancet Series | Improving access to analgesic drugs for patients with cancer in sub-Saharan Africa | 2013 | [https://www.theLancet.com/journals/lanonc/article/PIIS1470-2045(12)70343-1/fulltext](https://www.thelancet.com/journals/lanonc/article/PIIS1470-2045(12)70343-1/fulltext) |
| The Lancet Series | Every Newborn: health-systems bottlenecks and strategies to accelerate scale-up in countries | 2014 | [https://www.theLancet.com/journals/Lancet/article/PIIS0140-6736(14)60582-1/fulltext](https://www.thelancet.com/journals/lancet/article/PIIS0140-6736(14)60582-1/fulltext) |
| The Lancet series | Can available interventions end preventable deaths in mothers, newborn babies, and stillbirths, and at what cost? | 2014 | https://www.theLancet.com/journals/Lancet/article/PIIS0140-6736(14)60792-3/fulltext |
| The Lancet Commission | Global cancer surgery: delivering safe, affordable, and timely cancer surgery | 2015 | https://www.theLancet.com/journals/lanonc/article/PIIS1470-2045(15)00223-5/fulltext |
| The Lancet Commission | Global Surgery 2030: evidence and solutions for achieving health, welfare, and economic development | 2015 | [https://www.theLancet.com/journals/Lancet/article/PIIS0140-6736(15)60160-X/fulltext](https://www.thelancet.com/journals/lancet/article/PIIS0140-6736(15)60160-X/fulltext) |
| The Lancet Series | Improvement of fungal disease identification and management: combined health systems and public health approaches | 2017 | [https://www.theLancet.com/journals/laninf/article/PIIS1473-3099(17)30308-0/fulltext](https://www.thelancet.com/journals/laninf/article/PIIS1473-3099(17)30308-0/fulltext) |
| The Lancet Commission | The Lancet Commission on global mental health and sustainable development | 2018 | https://www.theLancet.com/journals/Lancet/article/PIIS0140-6736(18)31612-X/fulltext |
| The Lancet Series | Delivering modern, high-quality, affordable pathology and laboratory medicine to low-income and middle-income countries: a call to action | 2018 | https://www.theLancet.com/journals/Lancet/article/PIIS0140-6736(18)30460-4/fulltext |
| The Lancet Series | Improving pathology and laboratory medicine in low-income and middle-income countries: roadmap to solutions | 2018 | [https://www.theLancet.com/journals/Lancet/article/PIIS0140-6736(18)30459-8/fulltext](https://www.thelancet.com/journals/lancet/article/PIIS0140-6736(18)30459-8/fulltext) |
| The Lancet Commission | The Lancet NCDI Poverty Commission: bridging a gap in universal health coverage for the poorest billion | 2020 | [https://www.theLancet.com/journals/Lancet/article/PIIS0140-6736(20)31907-3/fulltext](https://www.thelancet.com/journals/lancet/article/PIIS0140-6736(20)31907-3/fulltext) |
| Academic publication (from pre-existing repository) | Which Surgical Operations Should be Performed in District Hospitals in East, Central and Southern Africa? Results of a Survey of Regional Clinicians | 2021 | <https://pubmed.ncbi.nlm.nih.gov/33000309/> |
| The Lancet Commission | The Lancet Commission on diagnostics: transforming access to diagnostics | 2021 | [https://www.theLancet.com/journals/Lancet/article/PIIS0140-6736(21)00673-5/fulltext](https://www.thelancet.com/journals/lancet/article/PIIS0140-6736(21)00673-5/fulltext) |
| Academic publication (from pre-existing repository) | Addressing severe chronic NCDs across Africa: measuring demand for the Package of Essential Non-communicable Disease Interventions-Plus (PEN-Plus) | 2022 | <https://www.ncbi.nlm.nih.gov/pmc/articles/PMC9006066/> |

**Supplementary Material 3: Policy documents included for data extraction**

| Source | Document | Link |
| --- | --- | --- |
| UNFPA | Managing Gender-based Violence Programmes in Emergencies: E-learning companion guide | https://www.unfpa.org/sites/default/files/pub-pdf/GBV E-Learning Companion Guide_ENGLISH.pdf |
| UNFPA | Women and Young Persons with Disabilities | https://www.unfpa.org/sites/default/files/pub-pdf/UNFPA-WEI_Guidelines_Disability_GBV_SRHR_FINAL_19-11-18_0.pdf |
| UNFPA | Adding It Up 2009: The Costs and Benefits of Investing in Family Planning and Maternal and Newborn Health | https://www.unfpa.org/sites/default/files/pub-pdf/adding_it_up_report.pdf |
| UNFPA | Maternal Death Surveillance and Response: Information For Action To Prevent Maternal Death | https://www.unfpa.org/sites/default/files/pub-pdf/9789241506083_eng.pdf |
| UNFPA | Guidance on Global Scale-Up of the Prevention of Mother-to-Child Transmission of HIV: Towards Universal Access for Women, Infants and Young Children and Eliminating HIV and AIDS Among Children | https://www.unfpa.org/sites/default/files/pub-pdf/prevention_hiv.pdf |
| WHO | Born too soon: decade of action on preterm birth | https://www.who.int/publications/i/item/9789240073890 |
| WHO | Universal access to safe blood transfusion | https://apps.who.int/iris/bitstream/handle/10665/69747/WHO_EHT_08.03_eng.pdf |
| WHO | Born too soon: decade of action on preterm birth | https://www.who.int/publications/i/item/9789240073890 |
| WHO | Guidance for national tuberculosis programmes on the management of tuberculosis in children | https://apps.who.int/iris/bitstream/handle/10665/112360/9789241548748_eng.pdf?sequence=1 |
| WHO | Packages of interventions: Family planning, safe abortion care, maternal, newborn and child health | https://www.who.int/publications/i/item/WHO-FCH-10.06 |
| WHO | WHO recommendations on the management of diarrhoea and pneumonia in HIV-infected infants and children Integrated Management of Childhood Illness (IMCI) | https://www.who.int/publications/i/item/9789241548083 |
| WHO | Buruli ulcer : diagnosis of Mycobacterium ulcerans disease. A manual for health care providers | https://www.who.int/publications/i/item/buruli-ulcer-diagnosis-of-mycobacterium-ulcerans-disease |
| WHO | Buruli ulcer : management of Mycobacterium ulcerans disease. A manual for health care providers | https://www.who.int/publications/i/item/who-cds-cpe-gbui-2001.3 |
| WHO | Congenital Rubella Syndrome: Vaccine Preventable Diseases Surveillance Standards | https://www.who.int/publications/m/item/vaccine-preventable-diseases-surveillance-standards-crs |
| WHO | Detection and investigation of serious adverse events following yellow fever vaccination | https://www.who.int/publications/i/item/detection-and-investigation-of-serious-adverse-events-following-yellow-fever-vaccination |
| WHO | Eradication of yaws – A guide for programme managers | https://www.who.int/publications/i/item/9789241512695 |
| WHO | Establishing syndromic surveillance and event-based surveillance systems for Zika, dengue and other arboviral diseases | https://www.who.int/publications/i/item/9789290223443 |
| WHO | Guidelines on Clinical Management of Chikungunya Fever | https://www.who.int/publications/i/item/guidelines-on-clinical-management-of-chikungunya-fever |
| WHO | Lymphatic filariasis: managing morbidity and preventing disability: an aide-mémoire for national programme managers, 2nd ed. | https://www.who.int/publications/i/item/9789240048294 |
| WHO | Malaria case management: operations manual | https://apps.who.int/iris/handle/10665/44124 |
| WHO | Overview: Vaccine Preventable Diseases Surveillance Standards | https://www.who.int/publications/m/item/vaccine-preventable-diseases-surveillance-standards_overview |
| WHO | Report of a WHO–FIND consultative meeting on diagnostics for Buruli ulcer | https://www.who.int/publications/i/item/WHO-HTM-NTD-IDM-2014.2 |
| WHO | Surveillance of adverse events following immunization against yellow fever | https://www.who.int/publications/i/item/WHO-HSE-GAR-ERI-2010-1 |
| WHO | Treatment of Mycobacterium ulcerans disease (Buruli Ulcer) Guidance for health workers | https://www.who.int/publications/i/item/9789241503402 |
| WHO | Mass casualty management systems : strategies and guidelines for building health sector capacity | https://www.who.int/publications/i/item/9789241596053 |
| WHO | Post-crash response: supporting those affected by road traffic crashes | https://www.who.int/publications/i/item/post-crash-response-supporting-those-affected-by-road-traffic-crashes |
| WHO | Save lives: a road safety technical package | https://www.who.int/publications/i/item/save-lives-a-road-safety-technical-package |
| WHO | Born too soon: decade of action on preterm birth | https://www.who.int/publications/i/item/9789240073890 |
| WHO | Packages of interventions: Family planning, safe abortion care, maternal, newborn and child health | https://www.who.int/publications/i/item/WHO-FCH-10.06 |
| WHO | Clinical management of rape and intimate partner violence survivors: developing protocols for use in humanitarian settings | https://www.who.int/publications/i/item/9789240001411 |
| WHO | Packages of interventions: Family planning, safe abortion care, maternal, newborn and child health | https://www.who.int/publications/i/item/WHO-FCH-10.06 |
| WHO | Strengthening health systems to respond to women subjected to intimate partner violence or sexual violence | https://www.who.int/publications/i/item/9789241513005 |
| WHO | Understanding and addressing violence against women: femicide | https://www.who.int/publications/i/item/WHO-RHR-12.38 |
| WHO | Guidelines on hepatitis B and C testing | https://www.who.int/publications/i/item/9789240008601 |
| WHO | IMAI district clinician manual: hospital care adolescents and adults | https://www.who.int/publications/i/item/imai-district-clinician-manual-hospital-care-adolescents-and-adults |
| WHO | Abortion care guideline | https://www.who.int/publications/i/item/9789240039483 |
| WHO | Born too soon: decade of action on preterm birth | https://www.who.int/publications/i/item/9789240073890 |
| WHO | District planning tool for maternal and newborn health strategy implementation A practical tool for strengthening Health Management System | https://www.who.int/publications/i/item/9789241500975 |
| WHO | Maintaining essential health services: operational guidance for the COVID-19 context: interim guidance, 1 June 2020 | https://www.who.int/publications/i/item/lymphatic-filariasis-managing-morbidity-and-preventing-disability-an-aide-m%C3%A9moire-for-national-programme-managers-2nd-ed |
| WHO | Managing complications in pregnancy and childbirth: A guide for midwives and doctors - Second Edition | https://www.who.int/publications/i/item/9789240001381 |
| WHO | Packages of interventions: Family planning, safe abortion care, maternal, newborn and child health | https://www.who.int/publications/i/item/WHO-FCH-10.06 |
| WHO | Born too soon: decade of action on preterm birth | https://www.who.int/publications/i/item/9789240073890 |
| WHO | Essential nutrition actions: mainstreaming nutrition through the life-course | https://www.who.int/publications/i/item/9789241515856 |
| WHO | Packages of interventions: Family planning, safe abortion care, maternal, newborn and child health | https://www.who.int/publications/i/item/WHO-FCH-10.06 |
| WHO | Protecting all against tetanus | https://www.who.int/publications/i/item/protecting-all-against-tetanus |
| WHO | Cancer control: Diagnosis and treatment. WHO guide for effective programmes | https://www.who.int/publications/i/item/9241547406 |
| WHO | CureAll framework: WHO global initiative for childhood cancer Increasing access, advancing quality, saving lives | https://apps.who.int/iris/handle/10665/347370 |
| WHO | Global breast cancer initiative implementation framework: assessing, strengthening and scaling up of services for the early detection and management of breast cancer | https://www.who.int/publications/i/item/9789240067134 |
| WHO | Preventing and controlling iron deficiency anaemia through primary health care: a guide for health administrators and programme managers | https://apps.who.int/iris/handle/10665/39849 |
| WHO | Tobacco and postsurgical outcomes | https://www.who.int/publications/i/item/9789240000360 |
| WHO | WHO framework for strengthening and scaling-up services for the management of invasive cervical cancer | https://www.who.int/publications/i/item/9789240003231 |
| WHO | Comprehensive cervical cancer control. A guide to essential practice - Second edition | https://www.who.int/publications/i/item/9789241548953 |
| WHO | WHO operational handbook on tuberculosis: module 4: treatment: tuberculosis care and support | https://www.who.int/publications/i/item/9789240053519 |
| WHO | Rehabilitation in health systems: guide for action | https://www.who.int/publications/i/item/9789241515986 |
| WHO | Surgical care at the district hospital | https://www.who.int/publications/i/item/9241545755 |
| WHO | Trachoma control: a guide for programme managers | https://www.who.int/publications/i/item/9241546905 |
| WHO | Chest radiography in tuberculosis detection: summary of current WHO recommendations and guidance on programmatic approaches | https://www.who.int/publications/i/item/9789241511506 |
| WHO | Consolidated guidelines on HIV prevention, testing, treatment, service delivery and monitoring: recommendations for a public health approach | https://www.who.int/publications/i/item/9789240031593 |
| WHO | Early detection of tuberculosis: an overview of approaches, guidelines and tools | https://www.who.int/publications/i/item/WHO-HTM-STB-PSI-2011.21 |
| WHO | Implementing the end TB Strategy | https://www.who.int/publications/i/item/implementing-the-end-tb-strategy |
| WHO | Management of MDR-TB : a field guide : a companion document to guidelines for programmatic management of drug-resistant tuberculosis : integrated management of adolescent and adult illness (‎IMAI)‎B | https://www.who.int/publications/i/item/9789241547765 |
| WHO | Packages of interventions: Family planning, safe abortion care, maternal, newborn and child health | https://apps.who.int/iris/handle/10665/70428 |
| WHO | WHO operational handbook on tuberculosis: module 3: diagnosis: rapid diagnostics for tuberculosis detection, 2021 update | https://www.who.int/publications/i/item/9789240030589 |
| WHO | Interagency List of Priority Medical Devices for Essential Interventions for Reproductive, Maternal, Newborn and Child Health | <https://www.who.int/publications/i/item/9789241565028> |
| WHO | The integrated management of adolescent and adult illness (IMAI) district clinician manual | <https://www.who.int/publications-detail-redirect/9789241548281> |
| WHO | Pocket book of hospital care for children: Second edition | <https://www.who.int/publications/i/item/978-92-4-154837-3> |
